# Clinical Sequelae Associated with Unresolved Tropical Splenomegaly in a Cohort of Recently Resettled Congolese Refugees in the United States—Multiple States, 2015–2018

**DOI:** 10.4269/ajtmh.19-0534

**Published:** 2020-05-04

**Authors:** Laura Divens Zambrano, Emily Jentes, Christina Phares, Michelle Weinberg, S. Patrick Kachur, Mukunda Singh Basnet, Alexander Klosovsky, Moses Mwesigwa, Marwan Naoum, Samuel Lubwama Nsobya, Olivia Samson, Matthew Goers, Robert McDonald, Bozena Morawski, Henry Njuguna, Corey Peak, Rebecca Laws, Yasser Bakhsh, Sally Ann Iverson, Carla Bezold, Hayder Allkhenfr, Roberta Horth, Jun Yang, Susan Miller, Michael Kacka, Abby Davids, Margaret Mortimer, William Stauffer, Nina Marano

**Affiliations:** 1 Epidemic Intelligence Service, CDC, Atlanta, Georgia;; 2 Division of Global Migration and Quarantine, National Center for Emerging and Zoonotic Infectious Diseases, CDC, Atlanta, Georgia;; 3 Columbia University Mailman School of Public Health, New York, New York;; 4 International Organization for Migration, Geneva, Switzerland;; 5 School of Biomedical Sciences, Makerere University College of Health Sciences, Kampala, Uganda;; 6 Infectious Disease Research Collaboration, Kampala, Uganda;; 7 Oak Ridge Institute for Science and Education, Oak Ridge, Tennessee;; 8 Division of Global Health Protection, Center for Global Health, CDC, Atlanta, Georgia;; 9 New York State Department of Health, Albany, New York;; 10 Idaho Department of Health and Welfare, Boise, Idaho;; 11 Washington State Department of Health, Tumwater, Washington;; 12 California Department of Public Health, Sacramento, California;; 13 Arizona Department of Health Services, Phoenix, Arizona;; 14 Utah Department of Health, Salt Lake City, Utah;; 15 Pennsylvania Department of Human Services, Harrisburg, Pennsylvania;; 16 South Carolina Department of Health and Environmental Control, Columbia, South Carolina;; 17 Family Medicine Residency of Idaho, Boise, Idaho;; 18 University of Minnesota, Minneapolis, Minnesota

## Abstract

Tropical splenomegaly is often associated with malaria and schistosomiasis. In 2014 and 2015, 145 Congolese refugees in western Uganda diagnosed with splenomegaly during predeparture medical examinations underwent enhanced screening for various etiologies. After anecdotal reports of unresolved splenomegaly and complications after U.S. arrival, patients were reassessed to describe long-term clinical progression after arrival in the United States. Post-arrival medical information was obtained through medical chart abstraction in collaboration with state health partners in nine participating states. We evaluated observed splenomegaly duration and associated clinical sequelae between 130 case patients from eastern Congo and 102 controls through adjusted hierarchical Poisson models, accounting for familial clustering. Of the 130 case patients, 95 (73.1%) had detectable splenomegaly after arrival. Of the 85 patients with records beyond 6 months, 45 (52.9%) had persistent splenomegaly, with a median persistence of 14.7 months (range 6.0–27.9 months). Of the 112 patients with available results, 65 (58.0%) patients had evidence of malaria infection, and the mean splenomegaly duration did not differ by *Plasmodium* species. Refugees with splenomegaly on arrival were 43% more likely to have anemia (adjusted relative risk [aRR]: 1.43, 95% CI: 1.04–1.97). Those with persistent splenomegaly were 60% more likely (adjusted relative risk [aRR]: 1.60, 95% CI: 1.15–2.23) to have a hematologic abnormality, particularly thrombocytopenia (aRR: 5.53, 95% CI: 1.73–17.62), and elevated alkaline phosphatase (aRR: 1.57, 95% CI: 1.03–2.40). Many patients experienced persistent splenomegaly, contradicting literature describing resolution after treatment and removal from an endemic setting. Other possible etiologies should be investigated and effective treatment, beyond treatment for malaria and schistosomiasis, explored.

## INTRODUCTION

Many diseases and conditions are associated with spleen enlargement or splenomegaly. The most common causes in the United States include liver disease, acute or chronic infections (e.g., bacterial endocarditis and infectious mononucleosis), hematologic malignancies (e.g., lymphomas, leukemias, and myeloproliferative disorders), inflammatory conditions (e.g., sarcoidosis, systemic lupus erythematosus, and rheumatoid arthritis), and splenic sequestration (e.g., pediatric sickle cell disease, hemolytic anemias, and thalassemias).^1^ Although these etiologies might affect populations from tropical areas, other potential causes include infectious etiologies. Tropical splenomegaly has been associated with malaria and B-cell lymphoproliferative disorders in a cohort of 221 Ghanaian patients,^2^ schistosomiasis in a case study describing a Sudanese refugee,^3^ hyperreactive malaria splenomegaly (HMS) syndrome among a cohort of Sudanese refugees,^4^ and hepatitis B virus infection, particularly in the presence of malaria infection, in a study of Hmong refugees.^5^

Splenomegaly is a common pathologic finding in the tropics. The most commonly reported etiology, HMS, is a diagnosis of exclusion. On-site malaria diagnostic testing is frequently negative in those with malaria-associated splenomegaly, possibly driven by the lower sensitivity of rapid diagnostic tests (RDTs) and smear microscopy relative to molecular diagnostic methods. In the absence of acute febrile illness and without a confirmatory test, malaria is assumed when other etiologies for splenomegaly have been excluded, and, generally, the individual responds to the treatment for malaria (e.g., decreased spleen size and decreased total IgM titers).^6^ Splenomegaly itself is associated with other morbidities, such as chronic hematologic and hepatic abnormalities and splenic rupture. Among a cohort of 381 Malawian preschool children, for example, anemia was significantly associated with both malaria and splenomegaly.^7^ A cohort of patients with hepatosplenic schistosomiasis in Brazil had elevated liver transaminases and alkaline phosphatase (ALP), increased risk of upper gastrointestinal bleeding, and hematologic sequelae, such as leukopenia, thrombocytopenia, anemia, and general pancytopenia, compared with controls. Authors found a significant inverse correlation between the longitudinal diameter of the spleen and platelet count.^8^

Approximately 6 months before refugees depart for the United States, U.S. Department of State–authorized panel physicians conduct required medical examinations according to technical instructions provided by the CDC.^9^ In 2014 and 2015, panel physicians working with the International Organization for Migration (IOM) in western Uganda notified CDC of an increase in the number of Congolese refugees with splenomegaly at their predeparture medical examinations. Under an enhanced screening protocol, IOM evaluated U.S.-bound Congolese refugees for splenomegaly in March and July 2015. Of the 987 persons screened during these two months, 145 (15%) had splenomegaly, defined as > 2 SDs above the mean maximum splenic length, adjusted for height, by the WHO ultrasonography protocol.^10^ Of these, 122 of 145 (64%) had massive splenomegaly, defined as > 4 SDs above the local mean for splenic size, adjusted for height.^10^ The results of this initial investigation are described elsewhere^11^; briefly, each individual enrolled in this initial investigation received further testing for malaria (thick and thin smear, RDT), schistosomiasis, leishmaniasis, hepatitis, tuberculosis, and sickle cell anemia.^11^ After this initial investigation, which only included RDT and thick- and thin-smear microscopy for malaria diagnostic testing, only 26.9% of refugees in this population tested positive for malaria, rendering little conclusive evidence to support a definitive infectious etiology. Assuming malaria was the most likely etiology, all patients presenting with splenomegaly at their predeparture medical examination during this period were presumptively treated with artemether–lumefantrine after the overseas medical examination and again just before departure for the United States. All patients also received praziquantel for presumptive treatment of schistosomiasis, in accordance with established treatment schedules for U.S.-bound refugees departing from sub-Saharan African countries.^12,13^ Of the 145 refugees, 143 resettled in 23 states across the United States between November 2015 and January 2016. CDC recommended that primaquine be provided after arrival in the United States to Congolese refugees with splenomegaly, after confirming normal levels of glucose-6-phosphate dehydrogenase (G6PD). Nested polymerase chain reaction (PCR) results, targeting 18S subunit ribosomal RNA, was used to identify *Plasmodium* species and was subsequently performed on all patients enrolled overseas using stored specimens.

Few data exist regarding the natural history or response to treatment of malaria-associated splenomegaly after a person is removed from an endemic area; only single cases or small case series have been reported. One recent review reported malaria-associated splenomegaly consistently resolves following short-course antimalarial treatment and removal from an endemic area.^6^ Another study of HMS reported that splenomegaly improved or resolved in 20 (91%) of 22 subjects who were followed up within 6 months. Most of these patients did not reside in endemic regions; of these 22 patients, four were immigrants and the rest were travelers from non-endemic countries.^14^ Based on this limited literature, it was expected that Congolese refugees with splenomegaly would have rapid resolution after arriving in the United States. However, in September 2016, CDC began receiving anecdotal clinical reports from clinicians in the United States of unresolved splenomegaly among refugees in the previously described cohort, despite antimalarial treatment (predeparture and after arrival) and removal from an endemic area. In addition, CDC received additional reports of complications among patients in this cohort, including one case of splenic rupture and a child who had died immediately on arrival to the United States from an unusual case of enterotoxigenic *Escherichia coli* sepsis, raising concerns that splenomegaly-associated immune-related complications may have contributed to his death.

Given that approximately 28,035 refugees from the Democratic Republic of the Congo resettled in the United States between January 2015 and December 2016, many refugees may have resettled with unrecognized splenomegaly. To inform clinical management of similar cases overseas and domestically, IOM asked CDC in April 2017 to initiate an investigation of unresolved splenomegaly in refugees of Congolese origin. The objective of this investigation was to improve our understanding of splenomegaly in individuals recently resettled from tropical and malaria-endemic areas, including clinical progression over time, associated clinical abnormalities, and, where possible, potential etiologies.

## MATERIALS AND METHODS

### Investigation design and participants.

**Figure 1.**
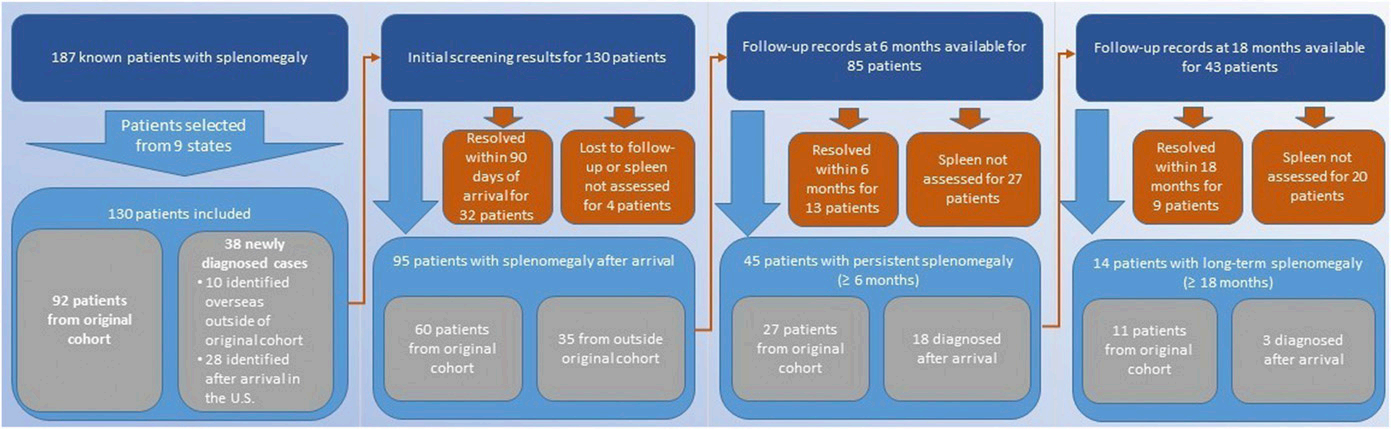
Flow diagram of inclusion of Congolese refugees for assessment of splenomegaly, duration of follow-up, and splenomegaly persistence after U.S. arrival (at arrival, 6 months after arrival, and 18 months after arrival) based on medical record review. The 187 patients with known splenomegaly include the 145 patients from the original cohort described by Goers et al.,^11^ of whom 92 resettled to the nine states included in this investigation. This investigation adds 38 new case patients not included in the original cohort. This figure appears in color at www.ajtmh.org.

Using information available for the 143 recently resettled refugees in the original cohort, we contacted the 10 states with the highest number of case patients with splenomegaly, plus Georgia; nine of these states agreed to participate.† We included a case cohort of 130 Congolese refugees who resettled between April 2015 and June 2017, comprising 92 of the 143 refugees from the original cohort^11,15^ who resettled to the nine participating states and any additional patients of Congolese origin with splenomegaly who were identified before departure to or after arrival in the United States (Figure 1). Specifically, we compared 130 splenomegaly case patients with matched controls to better characterize clinical sequelae associated with splenomegaly after arrival and to identify potential etiologies of splenomegaly in this population where data on potential exposures were available. Inclusion eligibility for the 92 patients who were included in the original overseas cohort was based on the case definition of splenomegaly according to the WHO ultrasonography protocol for schistosomiasis-related morbidity^10^; if spleen size (measured as the maximum length through the splenic hilus) exceeded 2 SD above the mean adjusted for height, a patient was eligible for enrollment.^10^ Case ascertainment for the 38 case patients identified after arrival was based solely on notes of splenomegaly in their medical records. Controls were identified from line lists of Congolese refugees who arrived from a resettlement camp in eastern or central Africa and were matched to case patients on age (within 2 years for children younger than 5 years, or within 5 years for children aged 5 years or older), gender, 12-month period of arrival, country of asylum (Uganda or Tanzania) before departure to the United States, and, if possible, U.S.-based initial screening clinic or, if not possible, state of initial screening clinic. As the objective of this investigation was to examine clinical progression and associated (predominantly hematologic) sequelae associated with unresolved splenomegaly, the sample size was selected to provide sufficient statistical power to detect a 24% difference in anemia prevalence between case patients and controls, based on estimates of splenomegaly and anemia prevalence among Malawian children^7^ and overall anemia prevalence among all people of Congolese origin.^16^ Methods for ascertaining sample size are described in detail in the supplementary material.

### Data collection.

Before departure, refugees with known splenomegaly underwent enhanced screening and diagnostic testing for multiple etiologies and associated conditions, such as malaria through thick- and thin-smear microscopy performed by a trained microbiologist and malaria Ag P.f./pan RDT (SD-BIOLINE, Lake Bluff, IL), *Schistosoma mansoni* through stool ova and parasite examination, leishmaniasis through rK39 RDT (SD-BIOLINE) tuberculosis, and viral hepatitis through detection of hepatitis B surface antigen (sAg) and hepatitis C (HCV) IgG (SD-BIOLINE HBsAg and HCV tests). International Organization for Migration personnel recorded data from this enhanced screening protocol on standard Department of State medical examination forms (DS-2054 and DS-3026); CDC personnel later entered information from these forms into the chart abstraction forms used for this investigation. In 2017, in response to the increase in U.S.-based clinician reports of unresolved splenomegaly among Congolese refugees, nested PCR for *Plasmodium* species diagnosis was performed following the protocol of Snounou et al.,^17^ at the Infectious Disease Research Collaboration Laboratories at Makerere University (Kampala, Uganda) on stored dried blood blotted on filtered paper of 144 of 145 patients in the original cohort, to enhance the sensitivity of parasite detection and to enable *Plasmodium* speciation. Among these 145 patients in the original cohort, 92 resettled to the states included in this investigation, 91 of whom had available PCR results, and neither predeparture nor post-arrival molecular results were generally available for patients identified outside the original cohort. Post-arrival medical information was obtained through medical records abstraction, and clinical information was recorded on abstraction forms designed for this investigation, in collaboration with state-level refugee health coordinators and local refugee health providers. Refugee health providers, state health department staff, or CDC staff performed chart abstractions in each of the nine states involved in this investigation and actively sought information from participating clinics on additional patients with splenomegaly among resettled Congolese refugees outside the original cohort at each clinic visited. All chart abstraction forms were centrally stored on a shared secure file transfer protocol site provided by the CDC.

Clinical indicators of interest included spleen size (as measured by palpation or ultrasound), splenomegaly duration, complete blood counts, liver function tests, and elevated total IgM. Current or previous malaria, tuberculosis, schistosomiasis, hepatitis, brucellosis, or Epstein–Barr virus infection; presence of intestinal parasites; and any other associated condition or potential exposure were recorded. Evidence of familial clustering was noted if more than one refugee under one Department of State refugee file number was noted to have splenomegaly. Post-arrival clinical data were collected from July 2017 to October 2018.

### Analysis.

Statistical analyses were performed in SAS V9.4 (Cary, NC). Differences in mean splenomegaly duration by infecting *Plasmodium* species were determined using a one-way analysis of variance test in patients who had follow-up records available for at least 6 months after their arrival to the United States. Hierarchical Poisson models were used to examine the association of persistent splenomegaly, defined as splenomegaly identified at or after arrival, and clinical sequelae of interest. All models used robust variance estimation to account for clustering among patients within families, and adjusted models controlled for the length of follow-up time, age, and gender. Controlling for the length of follow-up time accounted for the more limited longitudinal data available for the control population and for case patients who were lost to follow-up shortly after arrival.

### Ethics.

The protocol was approved by a human subjects advisor in the CDC National Center for Emerging and Zoonotic Diseases, who determined the investigation was a public health response and did not meet the definition of human subjects research that would be subject to Institutional Review Board (IRB) review. The human subjects review boards or IRBs of the Arizona Department of Human Services, the California Department of Public Health, the Georgia Department of Health, the Idaho Department of Health and Welfare, the New York State Department of Health, the Pennsylvania Department of Health, the South Carolina Department of Health and Environmental Control, the Utah Department of Health, and the Washington State Department of Social and Health Services each independently determined that the investigation was nonresearch. Confidentiality of all patient data collected for this investigation is strictly protected by an Assurance of Confidentiality under Section 308 (d) of the Public Health Service Act.

## RESULTS

### Description of case and control cohorts.

Investigators obtained medical records from initial screening clinics, primary care providers, and referral physicians for 130 case patients and 102 matched controls. Among the 130 case patients, 92 were from the original cohort,^11^ 10 were diagnosed overseas but were not part of the original cohort, and 28 were diagnosed after arrival in the United States (Figure 1). All patients included in this investigation resettled within the United States between April 1, 2015 and June 6, 2017; however, most of the case patients (105/130, 80.8%) resettled in the United States between April 1, 2015 and April 30, 2016, whereas the majority of the control patients (89/102, 87.3%) resettled across a broader date range, from April 1, 2015 to October 31, 2016. Most of the 130 case patients (*n* = 96, 73.5%) were younger than 25 years, 56 case patients (43.1%) were 15 years old or younger, and 77 case patients were male (59.2%) (Table 1). Similarly, most of the control patients (*n* = 78, 76.5%) were younger than 25 years, 44 controls (43.2%) were 15 years old or younger, and 61 (59.8%) of the controls were male (Table 1).

**Table 1:** Selected characteristics among Congolese refugees with splenomegaly and matched controls following resettlement to the United States

Characteristic	Cases (*n* = 130)	Controls (*n* = 102)	Total (*n* = 232)
	*N* (%)	*N* (%)	*N* (%)
Age on date of arrival (years)			
0 to < 5	3 (2.3)	2 (2.0)	5 (2.2)
5 to < 15	53 (40.8)	42 (41.2)	95 (40.9)
15 to < 25	40 (30.8)	34 (33.3)	74 (31.9)
25 to < 40	15 (11.5)	11 (10.8)	26 (11.2)
40 to < 60	16 (12.3)	11 (10.8)	27 (11.6)
60 and older	3 (2.3)	2 (2.0)	5 (2.2)
Gender			
Female	53 (40.8)	41 (40.2)	94 (40.5)
Male	77 (59.2)	61 (59.8)	138 (59.4)
Follow-up time (months)	(Mean: 12.7; SD: 9.2; range: 0.2–29.6)	(Mean: 9.7; SD: 10.4; range: 0–37.0]	(Mean: 11.4; SD: 9.8; range: 0–37.0)
≥ 6	85 (65.4)	45 (44.2)	130 (56.3)
≥ 18	43 (33.1)	22 (21.6)	65 (28.0)
In familial cluster*	87 (66.9)	37 (36.3)	143 (61.6)
Period of arrival			
December 1, 2014–March 31, 2015	0	3 (2.9)	3 (1.3)
April 1, 2015–October 15, 2015	38 (29.2)	22 (21.5)	60 (25.9)
October 16, 2015–April 30, 2016	67 (51.5)	34 (33.3)	101 (43.5)
May 1, 2016–October 31, 2016	13 (10.0)	33 (32.4)	46 (19.8)
November 1, 2016–June 6, 2017	12 (9.2)	10 (9.8)	22 (9.5)

* A familial cluster is defined as two or more refugees within either the case or control cohort who were in the same family.

### Splenomegaly persistence.

Predeparture and post-arrival medical and laboratory records were available for all 130 case patients, of whom 95 (73.1%) had detectable splenomegaly at their initial domestic screening visit (Figure 1). Among the 95 patients with detectable splenomegaly at arrival, 85 (89.5%) had medical records available beyond 6 months, 58 of whom had a recorded spleen examination; among these 58 patients, 45 (77.6%) had splenomegaly reported by physical examination or ultrasound more than 6 months after U.S. arrival, representing 52.9% of the patient population for whom any follow-up records were available for at least 6 months. After 18 months, 43 patients (45.3%) had medical records available, 23 of which included a spleen examination (Figure 1); among these 23 patients, 14 (60.9%) still had splenomegaly reported 18 months after U.S. arrival through the conclusion of this analysis. Among patients with persistent splenomegaly, the condition persisted for a median 9.4 months (interquartile range [IQR]: 2.7–15.9 months) and for an observed maximum of 27.9 months, although the condition could have persisted beyond the time frame of data collection.

### Additional case detection and familial clustering.

Among the 130 case-patients, 38 (29.2%) were identified outside the original cohort, 28 of whom were newly diagnosed after arrival and 10 of whom were diagnosed overseas. In addition, 87 (66.9%) were within family clusters with other splenomegaly case patients, and in some cases, proactive screening of family members of known case patients identified new cases. For example, clinicians in New York State proactively screened family members of known case patients using ultrasonography and identified six new patients with splenomegaly who were not identified through abdominal palpation.

### Laboratory results and treatment for investigated etiologies.

**Figure 2.**
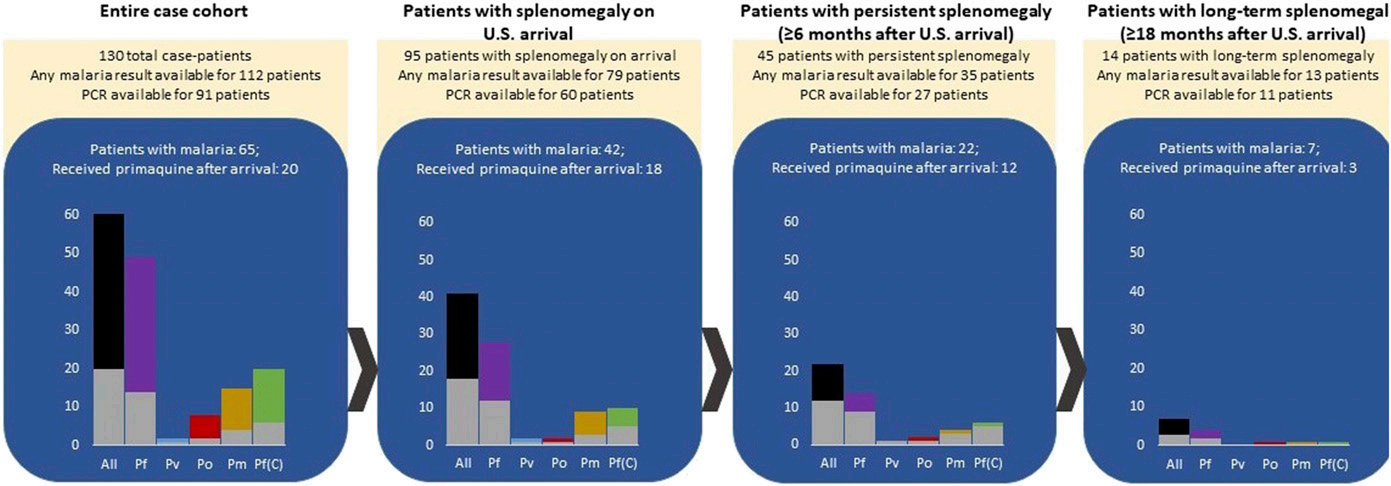
The proportion of Congolese refugees with splenomegaly with malaria attributable to any malaria species (black), *Plasmodium falciparum* (Pf, purple), *Plasmodium vivax* (Pv, blue), *Plasmodium ovale* (Po, red), *Plasmodium malariae* (Pm, gold), and coinfection with *P. falciparum* and another *Plasmodium* species (green) are shown, along with the proportion of those patients who received primaquine after arrival (gray). Malaria speciation was resolved through polymerase chain reaction among patients enrolled for enhanced screening by the International Organization for Migration at enrollment. This figure appears in color at www.ajtmh.org.

Although this investigation was designed only to evaluate and compare clinical sequelae and progression between case patients and controls after U.S. arrival, etiologic information was collected when possible. Information on potential exposures was not consistently available for cases and was particularly limited for controls, but all available etiologic information is presented in Table 2. Malaria microscopy, RDT, or PCR results from either predeparture or post-arrival visits were available for 112 (86.2%) of 130 cases, of whom 65 (58.0%) tested positive by any method (Table 2). Only 10 (9.8%) of 102 controls were tested for malaria, three of whom tested positive; testing methods among controls were restricted to microscopy and RDT. Malaria PCR results were available for 91 of the 92 patients who resettled to the nine states that participated. Among these 91 patients, 52 (57.1%) tested positive for any *Plasmodium* species by PCR, and of these, 20 patients had coinfections with multiple *Plasmodium* species, including *P. falciparum* coinfections with *Plasmodium malariae* (*n* = 12), *P. ovale* (*n* = 8), and *Plasmodium vivax* (*n* = 2), with two of these patients positive for three *Plasmodium* species. Of the 52 patients with any positive result, 49 (94.2%) tested positive for *P. falciparum*, 15 (28.8%) tested positive for *P. malariae*, eight (15.3%) tested positive for *P. ovale*, and two (3.8%) tested positive for *P. vivax* (Supplemental Table S2). Among the 45 refugees with persistent splenomegaly, 35 received testing for malaria, of whom 22 (62.9%) tested positive for malaria by any test (Supplemental Table S2, Figure 2). Of the 35 patients with persistent splenomegaly who received any malaria test, 27 patients received additional testing by PCR before departure. Of these 27 patients, 14 (51.9%) patients tested positive for *P. falciparum*, four (14.8%) tested positive for *P. malariae*, and three (11.1%) tested positive for *P. vivax* or *P. ovale*. Of the seven refugees with persistent splenomegaly who tested positive for non-*falciparum* malaria, five (71.4%) received the full course of recommended treatment, including primaquine (Supplemental Table S2, Figure 2). Mean splenomegaly duration did not differ by *Plasmodium* species or receipt of primaquine among patients who had follow-up records available at least 6 months after arrival (*P* = 0.632). Of all 130 case-patients, 48 (36.9%) received primaquine after arrival and 48 (36.9%) received primaquine after arrival and 28 (62.2%) of 45 refugees with persistent splenomegaly received primaquine at some point during follow-up. Malaria PCR results for all patients in the original cohort who underwent IOM’s enhanced screening protocol are provided in Supplemental Table S3.

**Table 2:** Results of laboratory screening for potential etiologies among Congolese refugees diagnosed with splenomegaly before or after U.S. resettlement compared with matched controls

Clinical characteristic*	All case patients (*n* = 130)		Splenomegaly on arrival (*n* = 95)		Persistent or chronic splenomegaly† (*n* = 45)		Splenomegaly not detected on arrival (*n* = 35)		Controls (*n* = 102)	
	*n*	%	*n*	%	*n*	%	*n*	%	*n*	%
Elevated IgM†	8/25	32.0	8/25	32.0	5/16	31.3	–	–	–	–
Malaria smear (+), polymerase chain reaction (+), or RDT (+)	65/112	58.0	42/79	53.2	22/35	62.9	23/32	71.9	3/10	30.0
Elevated *Schistosoma* IgG	17/30	56.7	16/28	57.1	11/17	64.7	1/2	50.0	4/12	33.3
Eosinophilia	9/16	56.3	9/15	60.0	6/10	60.0	0/1	0	0/3	0
No eosinophilia	7/16	43.8	6/15	40.0	4/10	40.0	1/1	100.0	3/3	100.0
Received primaquine§	48/129	37.2	45/94	47.9	28/45	62.2	3/35	8.6	–	–
Sickle cell anemia	0/53	0	0/33	0	0/19	0	0/20	0	–	–
Hepatitis A (IgG positive)	17/30	56.7	16/27	59.3	8/15	53.3	1/3	33.3	15/29	51.7
Hepatitis B (HBsAg positive)	1/115	0.9	1/84	1.2	1/40	2.5	0/31	0	2/98	2.0
Hepatitis C (IgG positive)	14/102	13.7	11/75	14.7	5/35	14.3	3/27	11.1	4/31	12.9

RDT = rapid diagnostic test; HBsAg = hepatitis B surface antigen.

* Two patients with splenomegaly were screened by serology for brucellosis, and neither was infected. No patients were screened for Epstein–Barr virus infection before departure or during the follow-up period. One patient tested positive for *Schistosoma mansoni* ova by stool ova and parasite examination before departure and had splenomegaly on arrival that resolved within 6 months.

† Persistent (splenomegaly present ≥ 6 months after arrival) or long-term splenomegaly (present ≥ 18 months after arrival) case patients are among those who had splenomegaly on arrival; that is, these are not mutually exclusive categories.

‡ Cutoff value for elevated total IgM: > 304 mg/dL. In the absence of cutoff values used for all domestic laboratories that screened these patients, the highest reported cutoff value was used.

§ Patient record of receipt of primaquine at any point after U.S. arrival, regardless of the duration of the course. AST = aspartate aminotransferase.

*Schistosoma* IgG ELISA results were available for 30 (23.1%) of 130 cases and 12 (11.8%) of 102 controls. Among the 17 case patients with persistent splenomegaly who had available *Schistosoma* IgG ELISA results, 11 (64.7%) were positive, and six (60.0%) of 10 patients with available paired absolute eosinophil counts had absolute eosinophilia (≥ 500 eosinophils/µL), possibly indicating ongoing schistosome infection. All patients in the original cohort received a stool ova and parasite examination, and one case patient included in this follow-up investigation tested positive for *S. mansoni* ova in addition to a second patient who was diagnosed with splenomegaly after U.S. arrival, although all patients (regardless of splenomegaly diagnosis) received preemptive treatment with praziquantel before departure for the United States. No additional O/P examinations were documented after U.S. arrival. Both patients had splenomegaly on arrival, although one patient was lost to follow-up 2 ½ months after arrival, and the condition resolved after 2 months in the other patient, who had 16 months of follow-up after arrival. Positive screening results for hepatitis A (IgG), hepatitis B (sAg), and HCV (IgG) had roughly equivalent proportions for both case patients and controls (Table 2). Of note, HCV serologies were unexpectedly high in both case patients and controls (14.7% and 13.7%, respectively). Sickle cell anemia was not detected in the 50 persons tested before departure. No patients were tested for Epstein–Barr virus infection (IgM or PCR), and only two patients were screened by serology for brucellosis, neither of whom were positive. No evidence of other autoimmune disorders, hemoglobinopathies, or cancers was documented in patients’ medical records.

### Associated comorbidities and complications.

Although clinical follow-up information varied in quality and duration across clinic sites, any refugee with splenomegaly at any point after arrival was 39% more likely to have had a hematologic abnormality, such as anemia, thrombocytopenia, or leukopenia (aRR: 1.39, 95% CI: 1.04–1.85), and, more specifically, was 43% more likely to have had anemia (aRR: 1.43, 95% CI: 1.04–1.97) (Table 3). Refugees with persistent splenomegaly beyond 6 months after arrival were 60% more likely (aRR: 1.60, 95% CI: 1.15–2.23) than controls to have had a hematologic abnormality at some point during the follow-up period. More specifically, patients with persistent splenomegaly were more than five times as likely to have had thrombocytopenia during the follow-up period (aRR: 5.53, 95% CI: 1.73–17.62). Splenomegaly did not appear to be associated with elevated liver transaminases (alanine transaminase and aspartate transaminase) in this population; however, refugees with persistent splenomegaly were 57% more likely to have elevated ALP (aRR: 1.57, 95% CI: 1.03–2.40). All estimates were adjusted for age, gender, and length of follow-up time.

**Table 3:** Comorbid conditions among Congolese refugees diagnosed with splenomegaly and their matched controls, measured at the initial post–U.S. arrival screening visit or the first available result

Condition*	Controls		Splenomegaly, all case patients		Crude RR (95% CI)	Adj RR (95% CI)
	*N*/Total	Proportion	*N*/Total	Proportion		
A Hematologic abnormality	35/80	0.44	78/129	0.60	1.33 (1.00–1.77)	1.28 (0.97–1.68)
Leukopenia	6/70	0.09	22/114	0.19	2.24 (0.97–5.14)	1.99 (0.84–4.70)
Anemia	30/79	0.38	65/128	0.51	1.32 (0.96–1.82)	1.30 (0.96–1.77)
Thrombocytopenia	2/21	0.10	30/101	0.30	3.07 (0.92–10.29)	2.98 (0.84–10.61)
Elevated transaminases	4/40	0.10	18/81	0.22	2.22 (0.86–5.74)	2.10 (0.77–5.68)
Elevated AST	0/40	0	15/81	0.19	–	–
Elevated ALT	4/40	0.10	11/81	0.14	1.39 (0.50–3.85)	1.24 (0.45–3.43)
Other						
Elevated ALP	20/39	0.51	47/82	0.57	1.07 (0.71–1.60)	1.17 (0.94–1.47)

Condition	Controls		Case patients with splenomegaly at intake		Crude RR (95% CI)	Adj RR (95% CI)
	*N*/Total	Proportion	*N*/Total	Proportion		
B Hematologic abnormality	35/80	0.44	61/94	0.65	1.44 (1.07–1.93)	1.39 (1.04–1.85)
Leukopenia	6/70	0.09	19/88	0.22	2.53 (1.10–5.84)	2.11 (0.86–5.21)
Anemia	30/79	0.38	50/93	0.54	1.41 (1.01–1.96)	1.43 (1.04–1.97)
Thrombocytopenia	2/21	0.10	24/74	0.32	3.41 (1.08–10.80)	3.18 (0.96–10.55)
Elevated transaminases	4/40	0.10	16/65	0.25	2.46 (0.95–6.36)	2.24 (0.82–6.14)
Elevated AST	0/40	0	14/65	0.22	–	–
Elevated ALT	4/40	0.10	9/65	0.14	1.41 (0.49–4.06)	1.22 (0.43–3.41)
Other						
Elevated ALP	20/39	0.51	36/65	0.55	1.02 (0.66–1.58)	1.21 (0.95–1.54)

Condition	Controls		Case patients with persistent splenomegaly (≥ 6 months)		Crude RR (95% CI)	Adj RR (95% CI)
	*N*/Total	Proportion	*N*/Total	Proportion		
C Hematologic abnormality	35/80	0.44	33/44	0.75	1.68 (1.28–2.20)	1.60 (1.15–2.24)
Leukopenia	6/70	0.09	13/41	0.32	3.72 (1.53–9.06)	2.00 (0.78–5.16)
Anemia	30/79	0.38	23/44	0.52	1.37 (0.96–1.95)	1.41 (0.93–2.16)
Thrombocytopenia	2/21	0.10	17/35	0.49	4.97 (1.39–17.83)	5.53 (1.73–17.62)
Elevated transaminases	4/40	0	8/35	0.23	2.40 (0.81–7.08)	2.98 (0.66–13.52)
Elevated AST	0/40	0.10	6/35	0.17	–	–
Elevated ALT	4/40		4/35	0.11	1.09 (0.27–4.44)	1.37 (0.35–5.37)
Other		0.51				
Elevated ALP	20/39	0.44	17/35	0.49	0.93 (0.54–1.60)	1.57 (1.03–2.40)

ALP = alkaline phosphatase; ALT = alanine aminotransferase. Relative risk of selected hematologic and hepatic abnormalities among Congolese refugees diagnosed with splenomegaly compared with their matched controls, measured at (**A**) any point during screening or follow-up, (**B**) at their initial post-arrival U.S. screening visit, and (**C**) 6 months or more after U.S. arrival.

* Cutoff vales: leukopenia (< 4.0 10^3^ cells/µL); anemia (males: < 14 g/dL; females: < 12.0 g/dL); thrombocytopenia (< 150 10^3^ PLT/µL); AST (≥ 40 U/L); ALT (≥ 40 U/L); ALP (≥ 147 U/L). In the absence of laboratory reference ranges from all domestic laboratories, the highest cutoff value reported was used, which might have sacrificed sensitivity.

One patient presented with massive splenomegaly (splenic size: 27.1 cm × 7.1 cm × 26 cm by ultrasound) and hepatomegaly with extensive thrombosis of the main portal vein and superior mesenteric and splenic veins. Although this patient did receive malaria treatment (artemether–lumefantrine) before departure, approximately 8 months after arrival, this patient required a splenectomy. Within one month of the splenectomy, this patient was hospitalized for clinical malaria. Clinical notes from this hospitalization did not indicate malaria testing methods or species, although *P. falciparum* was detected by PCR from the patient’s blood sample collected at her predeparture visit. The patient only initiated a 14-day course of primaquine to treat liver-stage hypnozoites after this hospitalization, not immediately after her arrival. There was no indication of any testing for schistosomiasis in this patient’s medical chart.

## DISCUSSION

This investigation adds to CDC’s previously published descriptive results of splenomegaly in individuals following departure from a tropical and malaria-endemic area.^11,15^ Persistent splenomegaly, defined as persistence beyond 6 months after arrival, was present in more than half of case-patients with available records; and chronic splenomegaly, defined as persistence beyond 18 months, occurred in about a third of patients with available follow-up records. Splenomegaly failed to resolve in many of the case-patients, despite previous literature suggesting the condition would improve in a vast majority of patients within the observed time frame of this investigation.^6,14^ These findings indicate that in certain populations, splenomegaly and associated morbidities will persist in a substantial proportion of individuals.

Despite previous treatment recommendations for malaria,^11,15^ only 36.9% of all case patients received primaquine after arrival to treat liver-stage hypnozoites; however, 60.9% of refugees with persistent splenomegaly received primaquine at some point during the follow-up, suggesting that it was more often considered for clinically complicated cases. Contributors to inconsistent primaquine administration may include the need for repeated visits for G6PD testing, a long (14-day) course, availability of primaquine, and lack of awareness among domestic physicians of CDC’s recommendation. Although inadequate treatment for malaria is not the sole contributor to splenomegaly persistence in this population, CDC has recommended that primaquine be prescribed to all refugees with splenomegaly arriving from a malaria-endemic region who are G6PD-negative, and clinical progression should be closely followed after treatment administration.

Refugees with splenomegaly on arrival were significantly more likely than matched controls to have a hematologic abnormality at some point during the follow-up, and the association was strengthened further when considering only case patients with persistent splenomegaly. Case patients appeared especially more likely to exhibit anemia and thrombocytopenia, likely due to splenic sequestration of blood cells and immune-mediated destruction of platelets through opsonization.^3,18^ Case patients were also significantly more likely to have elevated levels of ALP, which in this population, without associated other liver pathology described, likely represents a marker of poor nutritional status. These results, however, could be affected by the longer follow-up time and increased frequency of testing for case patients.

Although malaria-associated splenomegaly is a likely cause of splenomegaly in many of these patients, nearly 40% of patients without evidence of malaria infection had persistent splenomegaly (Supplemental Table S2). Future investigations should systematically collect detailed laboratory information to elucidate both infectious and noninfectious etiologies of splenomegaly in this population. Sickle cell anemia did not appear to contribute to splenomegaly in the group we assessed. Splenomegaly in this population may be multifactorial, and etiologies may have differed between patients. Evidence suggests that schistosomiasis may have contributed to persistent splenomegaly, as among patients with both available *Schistosoma* IgG ELISA and eosinophilia results, 60% tested positive for both; however, other etiologies for persistent eosinophilia exist and are common in this population, such as *Strongyloides*, filariasis (including *Loa loa*),^19^ and other parasitic infections (e.g., hookworm, trichuris, or tapeworm), which may not have responded completely to predeparture presumptive treatment. Only two refugees were tested for brucellosis, and none were tested for Epstein–Barr virus infection, which may both be associated with splenomegaly; future investigations should include testing for these two infections. In addition, further elucidation of the contribution and role of known chronic infections (e.g., viral hepatitis and schistosomiasis) and other hematologic and oncologic conditions, alone and in combination, should be further investigated. Of note, seroprevalence of anti-HCV IgG was unexpectedly high in case patients and controls, and although its contribution to splenomegaly is unknown, HCV seroprevalence was much higher than typically reported in other refugee populations.^20^ Although high seroprevalence of anti-HCV IgG has been reported in Congolese military members (13.7%),^21^ a population that would be expected to have higher risk, further investigation of HCV prevalence in Congolese refugees is encouraged.

Malaria PCR speciation indicated a higher than anticipated proportion of cases attributable to *P. malariae*, which may have diminished susceptibility to predeparture or subsequent antimalarial therapy with artemether–lumefantrine.^22^ A previous study of 2,588 samples from nine countries in sub-Saharan Africa using PCR indicated that *P. malariae* was represented in only 8.5% of all malaria infections^23^; however, among those with available molecular testing results in this investigation, 16.5% of all malaria infections were at least partially caused by *P. malariae* and 26.4% of infections were caused by multiple *Plasmodium* species. That said, *P. malariae–*associated malaria was less prevalent in persistent splenomegaly cases than in those with splenomegaly identified at intake only, although loss to follow-up of patients with splenomegaly could have contributed to this finding. Given available evidence that suggests splenomegaly may be associated with malaria, including *P. vivax* and *P. ovale*, in this cohort and from prior investigations,^11,14^ clinicians should administer primaquine to all eligible G6PD-negative patients with suspected tropical splenomegaly. Repeat treatment for schistosomiasis should be considered in clinically appropriate situations, such as persistent eosinophilia, although persistent eosinophilia can be attributed to other parasitic diseases, such as strongyloidiasis, filariasis, and roundworm infections.^19^ Given that the etiology of splenomegaly in this population is likely multifactorial, but most often involves malaria, clinicians should initiate clinical management using these guidelines while anticipating that individualized management will be needed. Clinicians treating suspected malaria-associated splenomegaly frequently follow total IgM levels to judge clinical response to antimalarial medication. Although this may be a useful clinical measure, total IgM was not tested in enough patients in this cohort to assess the clinical effectiveness of this strategy.

Considering the 28 cases identified at the initial domestic examination (and not identified overseas), increased awareness and emphasis on careful spleen examination may improve predeparture identification sensitivity, and domestic clinicians should remain (or become) vigilant. Given high familial clustering and additional cases identified through proactive family screening, both overseas and domestic clinicians should carefully screen family members by ultrasound, if possible, because clinical findings can be subtle and may be missed by palpation.

### Limitations.

Because we relied on data reported from multiple facilities on clinic visits that occurred at irregular intervals, this investigation likely underestimates the duration of splenomegaly. Given the nature of data collection through retrospective post-arrival chart abstraction, rather than prospective systematic cohort enrollment, splenomegaly duration could not be compared systematically between groups using more robust statistical methods (e.g., Kaplan–Meier survival analysis or proportional odds regression). In addition, it is not possible to ascertain whether splenomegaly was present when abdominal examinations were not noted or performed. Although cases were identified overseas using an enhanced screening protocol that included both abdominal palpation and ultrasound, domestic abdominal examinations were inconsistently performed and reported; thus, there may have been unreported cases, and persistence may be underestimated. We could only determine the last reported date of splenomegaly persistence, but duration could not be precisely ascertained as the condition likely persisted beyond the last reported date of splenomegaly detection. In Uganda, malaria smears were read by only one person, a trained microbiologist; however, subsequent molecular analyses of these patient samples likely mitigated any loss of sensitivity. In the United States, medical and diagnostic procedures differed by clinic; notably, some case patients were identified domestically by ultrasound, although this procedure was used inconsistently across clinic sites. Notably, this investigation sought to characterize clinical progression of patients with splenomegaly after U.S. arrival. Systematic collection of clinical and laboratory evidence of infectious and noninfectious etiologies was not possible given the retrospective nature of this investigation and the high degree of variability of information available in patient medical histories; this was further complicated among patients who did not undergo the enhanced IOM screening protocol before departure for the United States. A more thorough understanding of the distribution of potential etiologies in this population is vital to better inform patient management paradigms for refugee health providers. Future investigations should incorporate a case–control design at the point of overseas care to systematically characterize exposures in refugees presenting with splenomegaly.

## CONCLUSION

Splenomegaly persisted after arrival among many of these patients despite information available in the published literature and common belief that resolution would follow malaria treatment and removal from a malaria-endemic area. Associated hematologic conditions, particularly anemia and thrombocytopenia, were more common in refugees with persistent splenomegaly than controls included in this cohort. Clinicians caring for these patients at both predeparture and post-arrival visits should be aware of the apparent high prevalence of splenomegaly in Congolese refugees^11^ and assume a high pretest probability, particularly among family members of patients known to have splenomegaly. Patients identified with splenomegaly should be treated with primaquine, if eligible. Retreatment with praziquantel in patients with persistent, unexplained, eosinophilia or other signs or symptoms of schistosomiasis should be considered while considering other causes of eosinophilia. Patients should also be counseled on the condition and precautions (e.g., avoidance of contact sports), followed closely, and referred for specialty care if they fail to respond to treatment. Multiple etiologies of persistent splenomegaly in this population are possible, but there is likely a predominant underlying infection and immune response. Future investigations may further reveal associated pathologies and etiologies of tropical splenomegaly in this population.

## Supplemental tables

Supplemental materials

## Footnotes

† The eight states with high numbers of splenomegaly cases included in this investigation were Arizona, California, Idaho, New York, Pennsylvania, South Carolina, Utah, and Washington. Georgia was added because CDC is located there.
