# Supplementary material for: Clinical Sequelae Associated with Unresolved Tropical Splenomegaly in a Cohort of Recently Resettled Congolese Refugees in the United States—Multiple States, 2015–2018
Source: Am J Trop Med Hyg. 2020 May 4;103(1):485–93. doi: 10.4269/ajtmh.19-0534 (PMC7356405; doi:10.4269/ajtmh.19-0534)
Supplement: Supplementary file 1 [file tpmd190534.SD1.docx]

The following are supplemental materials and will be published online only

**Supplemental material**

**Sample Size**

Prevalence of a common complication of splenomegaly, anemia, was considered the primary outcome, although other hematologic and hepatic clinical outcomes associated with splenomegaly, such as thrombocytopenia, leukopenia, and elevated transaminases, were concurrently explored. Anemia was selected as the outcome of interest given the availability of literature on the relative prevalence of anemia in residents of sub-Saharan Africa with splenomegaly and those without splenomegaly, as well as the high likelihood that all refugees included in our assessment would have been tested for anemia. The relative prevalence of anemia among populations originating in the Democratic Republic of the Congo (DRC) with and without splenomegaly is not precisely known; however, in a population of children under 5 years old in Malawi, 63.7% and 29.8% of children with and without splenomegaly had severe anemia (Hb<5g/dL), respectively ^7^. These estimates were used to conservatively calculate our power to detect a difference in anemia prevalence among refugees with and without splenomegaly in the DRC, as 70.6% of the Congolese population was estimated to be affected by moderate-to-severe anemia (Hb<11g/dL) ^16^. Accounting for 20% loss to follow-up, a sample size of 90 refugees with splenomegaly in our original cohort and one matched control without splenomegaly yielded an overall sample size with 80% power to detect a difference in anemia prevalence of 63% vs. 39% among refugees with and without splenomegaly.

**Table S1**. Inclusion of patients by state in an investigation of splenomegaly among Congolese refugees resettled in the United States. Cases included those identified from the original overseas cohort, those identified overseas but outside of the original cohort, and those identified domestically.

|  | Cases diagnosed overseas | | Cases diagnosed domestically |  |  |  |
| --- | --- | --- | --- | --- | --- | --- |
| State | Member of original cohort * | Not a member of original cohort | Identified after arrival | Total number of cases included in this investigation | Number of cases with splenomegaly at initial exam | Number of cases with persistent splenomegaly |
| Arizona | 12 | 2 | 0 | 14 | 5 | 0 |
| California | 12 | 0 | 1 | 13 | 9 | 2 |
| Georgia | 1 | 2 | 0 | 3 | 3 | 0 |
| Idaho^*^ | 19 | 1 | 7 | 27 | 20 | 10 |
| New York | 12 | 1 | 9 | 22 | 18 | 8 |
| Pennsylvania | 11 | 1 | 0 | 12 | 4 | 1 |
| South Carolina | 6 | 0 | 7 | 13 | 11 | 9 |
| Utah | 13 | 3 | 2 | 18 | 18 | 11 |
| Washington | 6 | 0 | 2 | 8 | 7 | 4 |
| **Total** | **92** | **10** | **28** | **130** | **95** | **45** |

*Goers et al. (2016)

.

**Table S2.** Malaria testing results for Congolese refugees diagnosed with splenomegaly, including all testing that occurred before departure and after US arrival, and results of malaria PCR testing of refugees with splenomegaly on specimens collected before departure, categorized by duration of splenomegaly and treatment received.


*Malaria at any point during pre-departure screenings or post-arrival follow-up, by thick or thin smear microscopy, rapid diagnostic testing (RDT), or PCR.
^†^ Among the 102 control patients, only 10 had any record of malaria testing, and 3 patients tested positive for malaria by thick or thin smear microscopy or RDT. No molecular speciation data were available for control patients. Abbreviations: AM/LUM = artemether-lumefantrine, PQ = primaquine

**Table S3.** Malaria PCR results for all Congolese refugees with splenomegaly included in the cohort originally identified by the International organization for Migration (IOM)* through enhanced screening. The results are listed as a percentage of all patients included in this cohort (N=144). The first group represents ANY positive molecular result for any of the four Plasmodium species included in the screening. The second group, “Any combination infection,” represents a subset of the first group to describe the proportion of refugees in the original cohort infected with two or more Plasmodium species. The third group, “At least three species detected,” is a subset of the second group, and represents the proportion of refugees in the original cohort infected with at least three Plasmodium species. Numbers of affected patients by each pathogen or pathogen combination described is a subset of its group and will not add up to the total number of members in each group due to the presence of combination infections.

| **Malaria PCR results** | **N (%)** |
| --- | --- |
| **Relative prevalence of various *Plasmodium* species** |  |
| **Any positive molecular result** | **86 (59.7)** |
| *P. falciparum* | 83 (57.6) |
| *P. malariae* | 29 (20.1) |
| *P. ovale* | 12 (8.3) |
| *P.vivax* | 2 (1.4) |
| **Any combination infection** | **35 (24.3)** |
| *P. falciparum* + *P. malariae* | 26 (18.1) |
| *P. falciparum* + *P. ovale* | 12 (8.3) |
| *P. falciparum* + *P. vivax* | 2 (1.4) |
| **At least three species detected** | **5 (3.5)** |
| *P. falciparum* + *P. malariae* + *P. ovale* | 4 (2.8) |
| *P. falciparum* + *P. malariae* + *P. vivax* | 1 (0.7) |

*Goers, et al. (2016).
